# Gas Production, Digestibility and Efficacy of Stored or Fresh Plant Extracts to Reduce Methane Production on Different Substrates

**DOI:** 10.3390/ani10010146

**Published:** 2020-01-16

**Authors:** Abiodun Mayowa Akanmu, Abubeker Hassen, Festus Adeyemi Adejoro

**Affiliations:** Department of Animal and Wildlife Sciences, University of Pretoria, Private bag X20, Pretoria Hatfield 0028, South Africa; festus.adejoro@tuks.co.za

**Keywords:** medicinal plants, methane, volatile fatty acids, organic matter digestibility, lucerne, *Eragrostis curvula*

## Abstract

**Simple Summary:**

Medicinal plants possess the ability to mitigate methane production from ruminants. Long-term stability of these plant extracts are essential qualities to be able to replace other rumen modifiers. After one year of storage, plant secondary metabolites used in this study reduced methane production from low-quality forages, without adverse effects on feed digestibility in vitro.

**Abstract:**

Natural compounds such as plant secondary metabolites (PSM) can be used to replace antibiotic growth promoters as rumen modifiers. In this study, the effectiveness of stored and freshly extracted *Aloe vera* (AV), *Azadirachta indica* (AZ), *Moringa oleifera* (MO), *Jatropha curcas* (JA), *Tithonia diversifolia* (TD) and *Carica papaya* (CP) crude extract and monensin on in vitro gas and methane production, organic matter digestibility (IVOMD) and volatile fatty acids (VFA) were evaluated using a total mixed ration (TMR), lucerne or *Eragrostis curvula* substrates. Fresh extracts were processed from the same batch of frozen (−20 °C) plant material a few days before the trial while the stored extracts were extracted and stored at 4 °C for 12 months prior to the study. Extraction was done by solubilising 50 g freeze-dried plant material in 500 mL 100% methanol. Four mL of reconstituted 50 mg crude extract per 1000 mL distilled water was added per incubation vial, which already contained 400 mg substrate and in vitro fermentation, and gas production and IVOMD evaluation were carried out using standard procedures. Results showed that storing plant extracts for 12 months did not affect the activity or stability of metabolites present in the crude extracts, as shown by the lack of differences in total gas production (TGP) and methane produced between fresh or stored extracts across the substrates. In the TMR substrate, plant extracts increased IVOMD but did not affect TGP and methane production, whereas monensin did not have any effect. Plant extracts increased IVOMD of Eragrostis substrate and supressed methane production to a greater extent than monensin (*p* < 0.05). It can be concluded that storing plant extracts for up to 12 months did not compromise their efficacy. In addition, the use of 50 mg/kg of AV, AZ, MO, JA, TD and CP extract to a forage-based diet will reduce methane production while improving feed digestibility.

## 1. Introduction

Several studies have documented the potential benefits of medicinal plants and natural products for replacing antibiotic growth promoters in animal feeding [1,2,3]. The use of medicinal plant-based additives in the diet of farm animals poses little or zero threat to human beings who consume such products when used correctly. Previous studies [2,4,5,6] have revealed the beneficial effects of including medicinal plants to animals, such as in combating diseases, improving animal welfare and also increasing feed digestibility. Antibiotics commonly used for disease treatments and as growth promoters in animal agriculture are reported to have lengthy residence in animal products with its attendant food safety concerns in humans. One such food safety concern is antibiotic resistance syndrome in humans consuming animal products produced with antibiotics [7].

With limited food resources and stiffer competition with monogastrics, meeting the demand for animal protein may be achieved with improvement in the feed conversion efficiency of ruminant animals. However, in ruminants, about 6–15% of ingested energy meant for growth and production purposes is lost as methane through eructation [8,9,10]. Although methane production is one of the mechanisms that sustains normal rumen fermentation and fibre digestibility, significantly higher energy losses occur when animals consume poor quality roughages, as opposed to good quality roughages and concentrates. Aside from the loss of feed energy, rumen-derived methane is also a major anthropogenic source of greenhouse gases which results in global warming. It is estimated that about 18% of greenhouse gases worldwide are from agriculture sources, with ruminant animals accounting for the largest single source [11]. Therefore, effective methane mitigation has the potential to improve feed energy utilisation as well as reduce the environmental impacts of ruminant production.

*Aloe vera* (AV), *Azadirachta indica* (AZ), *Moringa oleifera* (MO), *Tithonia diversifolia* (TD), *Jatropha curcas* (JA) and *Carica papaya* (CP) have been reported to possess different phytochemicals such as anthraquinones, saponins, essential oils, catalase, azadirachtin, diastase and different digestive enzymes that could influence ruminal fermentation [1,2,12,13]. In a previous study [2], extracts of these plants, at 50 mg/kg substrate, reduced in vitro methane production and also increased the digestibility of Eragrostis hay, a low-quality roughage diet. Reducing methane production from ruminants without any adverse effect on feed digestibility is a ‘win-win’ situation for ruminants’ animal production. However, to validate the beneficial effect of these plant materials, it is necessary to test their effectiveness on a wide variety of feedstuffs with varying nutritional quality. Furthermore, it is not clear if the potency of these PSMs are retained over a prolonged storage period. Therefore, the aim of this study was to evaluate the effect of extract storage on the efficacy of these PSMs on in vitro gas and methane production, in vitro organic matter digestibility (IVOMD) and volatile fatty acids (VFA) production using a total mixed ration (TMR), lucerne or *Eragrostis curvula* as substrates. The substrates used in this study represent a high-quality feed TMR, high-quality forage (lucerne hay) and low-quality hay (eragrostis) available in Southern Africa.

## 2. Materials and Methods

### 2.1. Collection and Preparation of Plant Extracts

Foliage of AV, AZ, MO, TD, JA and CP were harvested from 10 different trees within the same farm in the South West region of Nigeria. The plant materials were rinsed with water to remove dirt, pests and foreign materials before transferring into a waiting refrigerating van. All procedures involved during collection, handling and transport of plant materials to the Department of Animal and Wildlife Sciences, University of Pretoria have been fully described [2]. Frozen plant materials were freeze-dried until a constant weight was achieved and milled through a 1 mm sieve. Extraction was done by adding 1500 mL 100% methanol to 150 g of dried plant materials. The mixture was then left for 4 days with periodic agitation to allow for thorough solubilisation into the solvent. The mixture was sieved through a 150 µm sieve and the filtrate was placed in a fume cupboard to evaporate excess methanol. To achieve complete dryness, the semi-dried plant extracts were later freeze-dried to a constant weight and a powdered product was recovered. Plant extract solutions of AV, AZ, MO, TD, JA and CP were prepared by reconstitution of 50 mg of each plant extract in 1000 mL distilled water to achieve the recommended dosage [2]. This solution was stored at 4 °C for a period of 12 months and designated as the stored extracts. After 12 months, a different set of plant extract was prepared and reconstituted from the same batch of plant materials that had been previously frozen at −20 °C.

### 2.2. Substrates and Chemical Analyses

The substrates used in this study were TMR, lucerne hay and eragrostis hay. TMR for feedlot lambs was purchased from a commercial feed mill in South Africa. All the substrates were analysed to determine their chemical composition in terms of dry matter (DM), total ash and ether extract using the Association of Official Analytical Chemists (AOAC) procedure [14]. Fibre fractions, which includes acid detergent fibre (ADF), neutral detergent fibre (NDF) and acid detergent lignin (ADL) of these substrates, were determined using ANKOM 200/220 technology (Macedon, NY, USA) as described by Van Soest et al. [15]. Crude protein was obtained by analysing the samples for nitrogen with Leco nitrogen/protein analyser (Leco Instrumente GmbH, Kirchheim, Germany).

### 2.3. In Vitro Gas, Methane and Organic Matter Digestibility

Two ruminally fistulated Merino sheep fed lucerne hay ad libitum were used as donor sheep throughout the experiment. Prior to rumen fluid collection, the animals were checked for intact cannula and signs of good health for 3 consecutive days. Ruminal fluid of about 900 mL was collected from each donor sheep before morning feeding and strained through four layers of cheese cloth into a pre-warmed thermos flask. Rumen fluid was transported to the laboratory and placed inside a water bath already heated to 39 °C with continuous flushing with CO_2_. A buffer mineral solution was prepared following a standard procedure [2]. Prior to incubation, 400 mg TMR, lucerne and eragrostis substrates had been weighed into 120 mL serum bottles and 4 mL of the stored or fresh plant extract solutions of AV, AZ, MO, TD, JA and CP was added 24 h before incubation. Monensin (Rumensin Elanco, Johannesburg, Gauteng, South Africa) was included as a positive control at a dose of 15 mg/kg DM feed, based on the manufacturer’s instruction. All stored and fresh extracts of each plant material, monensin and negative control (with 4 mL distilled water) groups were replicated 4 times within each incubation run and the experiment was repeated 5 times. Three blanks were included in each incubation run. All other procedures followed during the in vitro incubation procedure were as reported in a previous study [2].

### 2.4. Measurement of Total Gas, Methane, VFA and IVOMD

Gas pressure from each incubated serum bottle was measured at 3, 6, 12, 24 and 48 h after incubation using a pressure transducer PX4200-015GI (Omega Engineering Inc., Laval, QC, Canada) fitted with a digital data tracker (Tracker 220 series indicators; Omega Engineering Inc., Swedesboro, NJ, USA). The pressure transducer tip had been modified to fit into 3-way taps fitted on the serum bottles. All gas pressure readings were recorded in psi and subsequently converted to volume. Gas samples were also taken at each measurement time (3, 6, 12, 24, 48 h) for methane analysis. Methane concentration in the samples was obtained by injecting samples into a gas chromatograph (GC) (8610C, SRI Instruments GmbH, Bad Honnef, Germany) equipped with a flame ionisation detector. The GC had been pre-calibrated with 200, 500 and 1000 ppm standard methane. Methane concentration was corrected with gas volume at different collection times to estimate the volume of methane produced. Both gas and methane volume across the collection times were added to estimate the cumulative value up to 48 h. The incubation was terminated at 48 h by placing all bottles inside ice. The bottles were centrifuged at 4500× *g* for 15 min and 5 mL of the supernatant was collected and stored at −20 °C for VFA analysis [16]. In vitro organic matter digestibility was carried out on the residue using the two-stage digestion as described by Tilley and Terry [17] and modified by Engels and Van der Merwe [18].

### 2.5. Statistical Analyses

All data obtained were checked for normality using the PROC UNIVARIATE procedure and analysed using the general linear model of SAS 9.4 (SAS Institute Inc., Cary, NC, USA). Differences among means were compared using Tukey’s test of SAS. The experimental design was a completely randomised design. The statistical model used is listed below:

y_ijk_ = μ + Block/Run + P_i_ + S_j_ +(PS)_ij_ + εijk (1)

where y_ijk_ = observation k for different plant extract P (i; AV, AZ, MO, etc.) and level j of extract storage S (j; stored/old, fresh/new), μ = overall mean, block = effect of blocking (incubation runs), PS is the interaction effect between PSM and storage, µ is the overall mean and ε_ijk_ is the effect of random error.

## 3. Results and Discussion

The chemical analysis of the substrates used in this study is shown in Table 1. Crude protein levels of the substrates ranged between 56.1 g/kg and 195 g/kg DM. While both TMR and lucerne had high crude protein to sustain rumen fermentation, the CP value of eragrostis hay was lower than the 80 g/kg DM minimum required for optimal microbial biomass production, as noted by Ikhimioya [19]. The ADL fraction of all three substrates was low. The NDF fraction was highest in eragrostis substrate (784 g/kg), followed by lucerne (406 g/kg) and was least in the TMR substrate (301 g/kg) DM. Table 2 presents the total gas production (TGP), methane, IVOMD and TVFA when stored or fresh plant extracts of the medicinal plants were added into various substrates. There was no significant interaction between extract type and storage for TGP, methane and TVFA. Stored extracts of AV, AZ, MO, TD, JA and CP were as effective as the fresh extracts in terms of their effect on TGP, methane and total volatile fatty acid production for all the substrates used in this study.

Generally, storage seems not to have any significant effect on the efficacy of plant extracts. This suggests the stability of the plant extracts under storage, as tested in this study. The lack of differences could be due to the stabilising ability of the PSMs present in these extracts under the storage conditions. Equally, the method of extraction may have exerted minimal interference on bioactivity of the PSMs present. According to Mediani et al. [19], the activity of plant metabolites could decline over time from the onset of harvest, handling, drying, extraction or storage. The continuing activity of endoenzymes usually present in plant materials can reduce the free radical scavenging properties of PSMs. A possible interaction between phenol and polyphenol oxidase enzyme under the high-water activity of extracts in solution could result in reduced total phenolic content of plant extracts [20]. This may therefore impact rumen fermentation and substrate degradation. The result of this study is consistent with the previous study on *Cosmos caudatus*, where fresh or extracts stored for three months, from either air-dried or freeze-dried plants’ extracts, were compared. In that study, the storage or drying method did not affect the free radical scavenging activity of the extracts [21]. While the extracts in the report of Mediani et al. [21] were stored at −20 °C for three months, in the current study, aqueous extract solutions were stored at 4 °C for 12 months. Cold storage has been noted to halt enzymatic reactions that are capable of reducing the effectiveness of phytochemicals found in the plants [22]. It is likely that even under high-water activity of extracts in solution, cold storage can halt these enzymatic reactions. In a similar study [23], the antibacterial activity of different medicinal plants found in South Africa were retained in most of the species after 12 months of storage. Prolonged storage up to 12 years of dried plant materials in a dark cupboard has also shown that total phenolic content and inhibition of acetylcholinesterase by both fresh and stored plants were not significantly different [24].

There was a significant interaction effect between extract type and storage on IVOMD in the TMR substrate but not in the lucerne and eragrostis substrates. The simple effect of extract supplementation showed that extract type affected TGP, methane volume and TVFA in the eragrostis, lucerne and TMR substrates (*p* < 0.05). While inclusion of AZ, MO, TD and JA resulted in lower TGP compared to the TMR substrate, AV and CP inclusion did not affect TGP in the TMR substrate. Total gas production in the monensin treatment was not different from the control TMR substrate. In the lucerne substrate, while Mon, AV, JA, and CP were not different from the control, TGP was lower with AZ, MO and TD treatment. Meanwhile AZ, JA, MO, TD and CP produced lower TGP compared to the eragrostis-only substrate while monensin and AV treatments were not different from the eragrostis-only treatment. Methane volume was affected by extract type (*p* < 0.05) with all plant extracts and monensin supplementation resulting in lower methane compared to the TMR-alone substrate. While monensin did not affect methane volume in the lucerne substrate, AZ, MO, TD, JA and CP extract inclusion resulted in reduced methane production. All plant extracts reduced methane volume in the eragrostis hay substrate except monensin. While AV, AZ and MO resulted in higher IVOMD compared to the TMR substrate alone, TD, CP, Mon and CP also increased IVOMD but to a lesser extent. In the lucerne substrate, IVOMD was not affected by extract type. Inclusion of AV, MO, TD and CP extracts resulted in higher IVOMD compared to the extracts of AZ and JA, while monensin and the control treatments produced the least IVOMD in the eragrostis substrate. While fresh extract of AV and AZ produced higher IVOMD in the TMR substrate, storage did not affect the extract of MO, TD, JA and CP. In the TMR substrate, while TVFA was not affected by Mon, AZ, MO, TD, JA and CP, extract of AV resulted in higher TVFA. In the lucerne substrate, Mon resulted in lower TVFA while TD and JA extract increased TVFA production. In the eragrostis substrate, extract type did not affect TVFA production.

The efficacy of plant extracts may be considerably affected by the diet characteristics. Previous authors [25,26] have reported that ruminants consuming poor quality forage generally tend to produce higher methane intensity, while PSM supplementation may equally be affected by such factors like diet CP, rumen degradable nitrogen and dietary fibre characteristics. Furthermore, the report of Martinez et al. [27] indicated that a minimal effect was obtained in animal performance from the monensin-treated group when cows were fed a higher forage diet, and this may explain the lower response to plant extracts observed with the lucerne substrate, which has a high concentration of rumen degradable protein. In the eragrostis substrate, TGP and methane production were affected by the addition of plant extracts (*p* < 0.05) and the trend for each of the plant extracts was similar to previous results [2]. Total gas production for eragrostis hay alone was highest and this was followed by monensin and AV, while all the other plant extracts resulted in lower TGP (*p* < 0.05). Eragrostis is a drought-resistant grass with “midribs that exhibit a robust, lignified region of collenchyma cells (sclerenchyma) above the central vascular bundle and phloem fibres below” [28]. These properties give increased tensile strength and therefore, lower digestibility by ruminants. Methane production from the eragrostis substrate was significantly reduced by the inclusion of PSM (*p* < 0.01) regardless of the storage condition of the extract. When compared with the control diet, extracts of AV, AZ, MO, TD, JA and CP reduced methane by over 50%, while monensin reduced methane by 19%. Monensin, an antibiotic growth promoter, is effective in animals consuming high forage diets as it improves forage utilisation and feed efficiency. The significant reduction in methane witnessed with eragrostis hay could be attributed to the antimethanogenic properties of the PSMs present in the extracts, as previously noted by Akanmu and Hassen [2]. The interaction of different PSMs present in the same medicinal plant, with the diverse microbial population in the rumen might yield different results from the use of pure compounds, such as purified tannins or saponins [29], especially in terms of its methane mitigation potential. In the study by Singh et al. [30], aqueous extract blends from *Sapindus mukorossi*, *Ficus bengalensis* and Eucalyptus essential oil reduced methane concentration with increasing inclusion of plant extract blends in both oat hay and TMR substrate.

The increase in IVOMD of eragrostis by all the plant extracts used in this study confirm earlier findings [2] about the potential of PSM to improve forage digestibility. The presence of phytochemicals diastase and amylase present in the AV extract have been associated with improved feed digestibility in ruminants [2]. Furthermore, the breakdown of long chain polysaccharides could have been positively affected by the presence of anthraquinones, which is present at varying proportions in all the medicinal plants used in this study, as previously noted [2]. Both AZ and MO extracts have been reported to equally improve feed digestibility at moderate doses [31]. Furthermore, improved digestibility of the substrate has been associated with the presence of azadirachtin in AZ and the high concentration of alkaloids in MO [32]. Both azadirachtin and alkaloids exhibit selective antimicrobial and laxative properties [33]. While the plant extract blends reported in Signh et al. [30] did not affect total digestible dry matter in the oat hay substrate, there was a significant reduction in digestibility in the TMR substrate. Meanwhile, Cieslak et al. [34] also reported that *Vaccinium vitis idaea* extract reduced methane without compromising feed digestibility.

There was no significant interaction effect between substrate type and extract storage on CH_4_/TGP, CH_4_/IVOMD, TGP/IVOMD and CH_4_/TVFA for all substrates tested (Table 3). Equally, extract storage did not affect any of the parameters except TGP/IVOMD ratio in the TMR substrate (*p* < 0.05). Nevertheless, for all substrates, extract type had a significant effect on CH_4_/TGP and TGP/TVFA (*p* < 0.05). While extract type did not affect CH_4_/IVOMD and TGP/IVOMD in the lucerne substrate, there were significant effects in the TMR and eragrostis substrates. There was up to a 50% reduction in CH_4_/TGP with the plant extracts supplementation when tested on the eragrostis substrate. In contrast, both monensin and AV supplementation did not affect CH_4_/TGP in the lucerne hay and TMR substrates. Extract of AV significantly (*p* < 0.05) reduced CH_4_/IVOMD, TGP/IVOMD, CH_4_/TVFA and TGP/TVFA from TMR and eragrostis but not in lucerne. Extract of AZ also significantly reduced (*p* < 0.05) CH_4_/IVOMD and TGP/IVOMD in the TMR and lucerne substrates. Patra and Yu [35] reported the anti-methanogenic effect of some of the medicinal plants upon the consequent significant reduction in the volume of in vitro gas and methane production. The reduction in methane intensity as reflected in reduced CH_4_/TGP and CH_4_/IVOMD ratios reported in this study is consistent with previous studies on the antimethanogenic properties of TD. *Tithonia diversifoia* (TD) is reported to contain moderate concentrations of tannins, flavonoids and alkaloids, and supplementing a tropical grass with 20% TD foliage reduced the methanogenic bacteria and protozoa populations as a result of the PSMs inherent in the plant [36]. In that study, a significant defaunating effect, increased biodiversity of bacteria and ruminal cellulolytic fungi population was observed in the rumen. This, therefore, has the tendency to increase dry matter digestibility and potential reduction in methane emissions. Flavonoids are benzo-1-pyrone derivatives with anti-inflammatory, antioxidant and antimicrobial properties [37]. Kim et al. [38] reported the enteric methane reducing properties of flavonoid-rich medicinal plants as a result of reduced protozoal or methanogen numbers.

Table 4 presents the effect of stored or fresh plant extracts on in vitro VFA production from different substrates. The interaction effect between extract type and storage was not significant for all VFA molar proportions as well as the acetate:propionate (A/P) ratio. Except for the A/P ratio in eragrostis hay, storage did not affect the efficacy of plant extracts in terms of acetate, propionate, iso-butyrate, butyrate, iso-valerate and valerate molar proportions. However, plant extract type significantly affected the molar proportions of the individual VFAs as well as the A/P ratio (*p* < 0.05). All plant extracts reduced (*p* < 0.05) the acetate proportion of the TVFA produced, unlike monensin which was not different from the control treatment. Extract type did not affect propionate proportion in the lucerne substrate, unlike in the TMR and eragrostis substrates. All the plant extracts resulted in higher propionate proportion (*p* < 0.05) in the eragrostis substrate and higher valerate molar proportion in the TMR and lucerne substrates. This resulted in a reduced A/P ratio in both TMR and lucerne substrates. Monensin, however, did not have any significant effect on A/P ratio in the TMR and lucerne substrates but resulted in a lower A/P ratio in the eragrostis substrate. In the eragrostis substrate, while fresh extracts of MO, TD, JA and CP resulted in a lower A/P ratio, stored extracts had no such effect. Storage did not affect AV and AZ in terms of its effect on the A/P ratio in the eragrostis substrate. Monensin did not have any effect on acetate and propionate proportion in the three substrates.

The reduction in A/P ratio associated with the inclusion of PSMs are usually due to reduced acetate, an increase in propionate or a shift from acetate to propionate. An increase in propionate observed in this study is similar to the reports of Adejoro et al. [39] on tannin, Cieslak et al. [34] on *Vaccinium vitis* extract supplementation and Wang et al. [12] on Atractylodes rhizome and Amur cork tree supplementation. Propionate formation and methanogenesis are competitive pathways for hydrogen metabolism in the rumen [34]. Methanogens utilise hydrogen in the rumen, and in the process, compete with propionate-producing microbes that also utilise hydrogen to form propionate [12]. Therefore, processes that enhance increased propionate could result in reduced methanogenesis, as shown by the results of this study. A lower A/P ratio associated with tannin is usually due to a reduction in the activities of acetate forming bacteria which result in an increased propionate proportion [34]. Acetate reduction due to supplementation of PSMs could also be due to a direct reduction in the activities of acetate forming bacteria or a reduction in hydrogen production [39,40]. Reduced hydrogen production will result in decreased fibre digestion. Nevertheless, reduced acetate did not result in decreased IVOMD in the current study.

## 4. Conclusions

Generally, extracts of *Aloe vera*, *Azadirachta indica*, *Carica papaya*, *Moringa oleifera*, *Jatropha curcas* and *Tithonia diversifolia* were potent methane reducing agents and storage for 12 months did not affect their potency, indicating that the bioactivity of the PSMs inherent in them did not deteriorate. Although the responses of the extracts varied due to substrate differences, these plant extracts effectively reduced methane emission from poor quality roughage (eragrostis) to a higher degree compared to lucerne and TMR substrates without a significant reduction in in vitro organic matter digestibility.

## Figures and Tables

**Table 1:** Chemical composition of *Eragrostis curvula* hay, lucerne hay and total mixed ration substrates used for in vitro incubation.

Composition in DM (g/kg DM)	TMR	Lucerne	Eragrostis
Crude protein	192	185	56.1
NDF	301	406	784
ADF	214	321	492
ADL	48	55	78
Ether extract	59	19	12
Ash	78	76	45

TMR, total mixed ration; NDF, neutral detergent fibre; ADF, acid detergent fibre; ADL, acid detergent lignin

**Table 2:** Total gas production (TGP), methane, in vitro organic matter digestibility (IVOMD) and total volatile fatty acid (TVFA) from *Eragrostis curvula*, lucerne and TMR substrates incubated with stored or fresh plant extracts.

Parameters	Substrates	Extracts	Cntrl	Mon	AV	AZ	MO	TD	JA	CP	SEM	*p*-Values		
												*p*	S	p*S
TGP, mL	TMR	Stored	89.6	86.2	85.6	85.1	83.4	80.4	82.1	85.6	0.59	0.02	0.53	0.91
		Fresh	89.6	86.2	83.4	81.5	82.6	82.3	82.0	85.9				
	Lucerne	Stored	63.8	56.2	60.1	57.3	56.2	58.2	62.8	61.1	0.49	<0.01	0.61	0.29
		Fresh	63.8	56.2	60.4	55.1	59.6	57.8	59.4	60.8				
	Eragrostis	Stored	55.3	53.6	54.2	49.7	50.8	49.2	47.3	50.1	0.58	<0.01	0.06	0.72
		Fresh	55.3	53.6	54.4	47.9	47.7	48.5	45.7	48.7				
Methane, mL	TMR	Stored	30.6	25.4	22.7	26.7	24.8	22.3	25.1	22.6	0.51	0.01	0.96	0.85
		Fresh	30.6	25.4	24.1	24.2	24.4	25.3	25.4	22.1				
	Lucerne	Stored	22.7	21.1	23.4	18.0	17.8	15.8	20.1	18.6	0.41	0.01	0.37	0.29
		Fresh	22.7	21.1	21.8	19.8	20.1	18.2	18.3	19.2				
	Eragrostis	Stored	17.9	14.5	8.72	9.22	7.72	7.87	7.18	6.56	0.76	<0.01	0.20	0.23
		Fresh	17.9	14.5	8.88	7.91	7.25	5.81	5.87	7.63				
IVOMD, %	TMR	Stored	71.1	76.1	80.8	77.2	77.5	76.1	73.4	76.4	0.76	<0.01	<0.01	0.01
		Fresh	71.1	76.1	88.2	83.1	80.3	79.8	77.3	77.7				
	Lucerne	Stored	61.6	68.6	63.4	63.7	59.6	64.4	62.5	64.7	1.55	0.22	0.35	0.49
		Fresh	61.6	68.6	62.5	63.1	63.7	62.7	61.9	64.2				
	Eragrostis	Stored	33.6	35.4	44.6	41.5	44.4	42.5	40.2	42.8	0.72	<0.01	0.42	0.97
		Fresh	33.6	35.4	45.8	41.8	45.1	42.6	41.5	42.3				
TVFA, mmol/L	TMR	Stored	121	115	129	117	120	118	119	117	0.67	<0.01	0.18	0.08
		Fresh	121	115	127	117	119	117	118	119				
	Lucerne	Stored	95.9	91.8	96.9	93.9	94.7	96.4	97.9	94.2	0.36	<0.01	0.98	0.89
		Fresh	95.9	91.5	96.9	93.1	94.8	97.2	97.5	94.5				
	Eragrostis	Stored	82.0	76.1	78.8	77.1	80.1	78.9	78.9	79.0	0.31	0.07	0.42	0.48
		Fresh	82.0	76.1	78.7	77.8	78.1	78.5	78.0	80.1				

Cntrl, control; Mon, monensin; AV, Aloe vera; AZ, Azadirachta indica; MO, Moringa oleifera; TD, Tithonia diversifolia; JA, Jatropha curcas; CP, Carica papaya; SEM, standard error of mean; *p*, plant extracts; S, storage; p*S, Interaction between plant extracts and storage.

**Table 3:** The ratios of total gas production (TGP), methane (CH_4_) and in vitro organic matter digestibility (IVOMD) from *Eragrostis curvula*, lucerne and TMR substrates incubated with stored or fresh plant extracts.

Parameters	Substrates	Extracts	Cntrl	Mon	AV	AZ	MO	TD	JA	CP	SEM	*p*-Values		
												*p*	S	p*S
CH_4_/TGP	TMR	Stored	34.1	29.5	26.5	31.4	29.5	30.7	30.5	26.4	0.49	0.01	0.71	0.88
		Fresh	34.1	29.5	28.6	29.7	29.4	28.5	30.9	25.7				
	Lucerne	Stored	35.5	34.7	38.9	31.4	31.2	27.3	32.1	30.4	0.58	0.01	0.22	0.28
		Fresh	35.5	34.7	36.0	36.1	33.6	31.5	30.8	31.7				
	Eragrostis	Stored	28.9	33.5	16.1	18.6	15.1	15.9	15.2	13.1	1.29	<0.01	0.31	0.25
		Fresh	28.9	33.5	16.3	16.5	15.3	12.0	12.9	15.7				
CH_4_/IVOMD, mL/kg	TMR	Stored	42.9	33.4	28.1	34.7	31.9	30.2	34.2	29.7	0.87	<0.01	0.21	0.71
		Fresh	42.9	33.4	27.2	29.1	30.3	31.6	32.8	28.3				
	Lucerne	Stored	36.8	30.7	36.9	28.3	29.9	24.6	32.2	28.8	3.52	0.23	0.29	0.50
		Fresh	36.8	30.7	34.7	31.6	31.5	29.1	29.6	30.1				
	Eragrostis	Stored	47.7	51.0	19.8	22.2	17.4	18.5	17.9	15.4	2.50	<0.01	0.20	0.25
		Fresh	47.7	51.0	19.4	18.9	16.1	13.6	14.1	18.0				
TGP/IVOMD, mL/kg	TMR	Stored	1.26	1.13	1.06	1.10	1.08	1.07	1.11	1.12	0.02	<0.01	0.02	0.18
		Fresh	1.26	1.13	0.95	0.97	1.03	1.02	1.06	1.10				
	Lucerne	Stored	1.04	0.88	0.94	0.89	0.94	0.91	1.00	0.94	0.09	0.32	0.35	0.48
		Fresh	1.04	0.8	0.96	0.87	0.93	0.92	0.95	0.94				
	Eragrostis	Stored	1.65	1.52	1.22	1.19	1.14	1.16	1.18	1.17	0.04	<0.01	0.15	0.96
		Fresh	1.65	1.52	1.19	1.15	1.06	1.14	1.09	1.15				
CH_4_/TVFA	TMR	Stored	25.2	22.0	17.5	22.7	20.7	19.3	21.1	19.4	0.43	<0.01	0.91	0.78
		Fresh	25.2	22.1	18.9	20.5	20.4	21.5	21.4	18.5				
	Lucerne	Stored	23.6	23.0	24.1	19.2	18.8	16.4	20.5	19.7	0.44	<0.01	0.38	0.37
		Fresh	23.6	23.1	22.5	21.3	21.1	18.7	18.8	20.4				
	Eragrostis	Stored	21.9	19.1	11.1	10.6	9.65	9.98	9.11	8.31	0.96	<0.01	0.29	0.26
		Fresh	21.9	19.1	11.2	10.1	9.26	7.41	7.52	9.51				
TGP/TVFA	TMR	Stored	73.9	74.8	66.1	72.3	69.8	67.9	69.0	73.3	0.59	<0.01	0.70	0.85
		Fresh	73.9	74.8	66.0	68.8	69.3	70.2	69.3	72.2				
	Lucerne	Stored	66.6	66.4	61.9	61.1	59.3	60.3	64.2	64.8	0.55	<0.01	0.66	0.54
		Fresh	66.6	66.4	62.3	59.2	62.8	59.5	60.9	64.3				
	Eragrostis	Stored	67.3	70.5	68.7	66.5	63.5	62.3	60.1	63.4	0.73	<0.01	0.06	0.64
		Fresh	67.3	70.5	69.1	61.6	61.1	61.7	58.5	60.7				

Cntrl, control; Mon, monensin; AV, Aloe vera; AZ, Azadirachta indica; MO, Moringa oleifera; TD, Tithonia diversifolia; JA, Jatropha curcas; CP, Carica papaya; SEM, standard error of mean; *p*, plant extracts; S, storage; p*S, Interaction between plant extracts and storage.

**Table 4:** Molar proportions of volatile fatty acids from the in vitro incubation of *E. curvula*, lucerne and TMR substrates from *E. curvula*, lucerne and TMR substrates incubated with stored or fresh plant extracts.

VFA Molar Proportions		Extracts	Cntrl	Mon	AV	AZ	MO	TD	JA	CP	SEM	*p*-Values		
												*p*	S	p*S
Acetate	TMR	Stored	45.5	44.5	33.6	33.9	31.3	31.5	33.5	33.2	1.00	<0.01	0.38	0.47
		Fresh	45.5	44.5	34.1	35.3	30.6	29.7	32.5	32.2				
	Lucerne	Stored	43.3	41.9	37.3	34.1	33.1	36.5	32.6	36.1	0.66	<0.01	0.95	0.34
		Fresh	43.3	41.9	35.4	35.5	34.1	34.5	34.6	35.6				
	Eragrostis	Stored	44.4	42.2	35.8	36.2	38.2	39.1	38.5	38.1	0.55	<0.01	0.26	0.28
		Fresh	44.4	42.0	37.6	36.1	39.0	37.9	34.5	36.8				
Propionate	TMR	Stored	22.7	23.2	20.3	20.2	21.3	19.6	19.7	21.8	0.32	0.04	0.62	0.92
		Fresh	22.7	23.2	20.3	19.9	22.6	21.8	18.9	21.6				
	Lucerne	Stored	21.0	19.4	20.8	20.3	19.7	20.6	19.6	20.7	0.13	0.82	0.60	0.14
		Fresh	20.1	20.9	19.4	19.7	19.4	20.2	20.7	19.6				
	Eragrostis	Stored	17.9	20.1	21.9	22.1	21.4	21.5	22.5	21.6	0.30	0.01	0.25	0.49
		Fresh	17.9	20.1	22.7	21.7	22.9	23.3	21.8	22.6				
Iso-butyrate	TMR	Stored	2.92	3.97	7.66	6.57	6.46	7.42	7.06	9.26	0.43	<0.01	0.79	0.19
		Fresh	2.92	3.97	5.80	4.72	6.84	8.63	10.2	9.21				
	Lucerne	Stored	3.00	13.1	8.3	9.59	8.98	7.51	9.16	8.62	0.49	<0.01	0.82	0.86
		Fresh	3.00	12.5	9.69	9.14	7.39	7.83	8.51	9.28				
	Eragrostis	Stored	4.89	5.91	12.5	8.97	9.39	9.19	8.63	9.35	0.40	<0.01	0.52	0.10
		Fresh	4.89	5.91	7.54	9.26	10.3	8.42	9.96	10.1				
Butyrate	TMR	Stored	12.8	14.5	18.9	19.7	20.3	20.4	19.4	18.5	0.51	<0.01	0.32	0.94
		Fresh	12.8	14.6	19.1	20.6	20.7	21.4	18.8	19.5				
	Lucerne	Stored	15.8	10.9	13.5	14.1	14.6	14.2	15.6	13.9	0.27	<0.01	0.68	0.62
		Fresh	15.8	10.4	14.1	14.3	14.8	14.7	14.8	14.1				
	Eragrostis	Stored	15.9	12.1	8.72	12.1	11.1	11.4	11.1	11.4	0.40	0.03	0.15	0.18
		Fresh	15.6	12.1	14.7	11.6	9.7	11.6	11.6	13.7				
Iso-valerate	TMR	Stored	11.7	9.52	10.3	10.1	11.1	11.3	10.5	11.1	0.16	0.04	0.65	0.99
		Fresh	11.7	9.52	1.1	10.6	11.1	11.1	10.5	11.1				
	Lucerne	Stored	12.9	8.16	10.1	10.8	12.1	10.8	12.3	10.3	0.33	0.01	0.60	0.95
		Fresh	12.9	12.8	11.3	10.8	12.9	11.8	11.2	11.1				
	Eragrostis	Stored	11.9	10.7	9.91	10.7	9.79	9.14	9.43	9.81	0.24	0.45	0.23	0.81
		Fresh	11.9	10.7	9.25	10.9	10.8	11.2	11.5	9.56				
Valerate	TMR	Stored	4.06	4.21	9.13	9.34	9.50	9.82	9.87	6.83	0.43	<0.01	0.21	0.80
		Fresh	4.05	4.21	9.6	8.99	8.13	7.51	9.14	6.58				
	Lucerne	Stored	4.76	4.72	10.0	11.1	11.7	10.2	10.6	10.4	0.47	<0.01	0.42	0.76
		Fresh	4.76	4.72	10.0	10.4	11.4	10.9	10.1	10.2				
	Eragrostis	Stored	5.22	6.61	11.2	10.0	10.2	9.65	9.86	9.81	0.43	0.03	0.19	0.71
		Fresh	5.22	6.61	8.16	10.4	7.64	7.52	11.3	7.61				
A: *p* ratio	TMR	Stored	1.99	1.92	1.65	1.67	1.46	1.60	1.69	1.52	0.04	<0.01	0.63	0.81
		Fresh	1.99	1.92	1.67	1.79	1.36	1.39	1.71	1.48				
	Lucerne	Stored	2.16	2.17	1.78	1.67	1.68	1.76	1.66	1.75	0.03	<0.01	0.74	0.45
		Fresh	2.16	2.17	1.82	1.79	1.75	1.71	1.67	1.81				
	Eragrostis	Stored	2.48	2.02	1.63	1.64	1.78	1.82	1.71	1.76	0.05	<0.01	0.03	0.42
		Fresh	2.48	2.02	1.65	1.66	1.70	1.63	1.63	1.60				

Cntrl, control; Mon, monensin; AV, Aloe vera; AZ, Azadirachta indica; MO, Moringa oleifera; TD, Tithonia diversifolia; JA, Jatropha curcas; CP, Carica papaya; SEM, standard error of mean; *p*, plant extracts; S, storage; p*S, Interaction between plant extracts and storage.

## References

[1] Menale B., De Castro O., Cascone C., Muoio R. (2016). Ethnobotanical investigation on medicinal plants in the Vesuvio National Park (Campania Southern Italy). J. Ethnopharmacol..

[2] Akanmu A.M., Hassen A. (2018). The use of certain medicinal plant extracts reduced in vitro methane production while improving in vitro organic matter digestibility. Anim. Prod. Sci..

[3] Adejoro F.A., Hassen A., Akanmu A.M. (2019). Effect of lipid-encapsulated acacia tannin extract on feed intake, nutrient digestibility and methane emission in sheep. Animals.

[4] Ramiah S.K., Zulkifli I., Rahim N.A.A., Ebrahimi M., Meng G.Y. (2014). Effects of two herbal extracts and virginiamycin supplementation on growth performance, intestinal microflora population and fatty acid composition in broiler chickens. Asian-Australas. J. Anim. Sci..

[5] Jahromi M.F., Altaher Y.W., Shokryazdan P., Ebrahimi R., Ebrahimi M., Idrus Z., Tufarelli V., Liang J.B. (2016). Dietary supplementation of a mixture of Lactobacillus strains enhances performance of broiler chickens raised under heat stress conditions. Int. J. Biometeorol..

[6] Lee S.J., Kim D.H., Guan L.L., Ahn S.K., Cho K.W., Lee S.S. (2015). Effect of medicinal plant by-products supplementation to total mixed ration on growth performance carcass characteristics and economic efficacy in the late fattening period of hanwoo steers. Asian-Australas. J. Anim. Sci..

[7] Mama O.M., Gómez P., Ruiz-Ripa L., Gómez-Sanz E., Zarazaga M., Torres C. (2019). Antimicrobial Resistance, Virulence, and Genetic Lineages of Staphylococci from Horses Destined for Human Consumption: High Detection of S. aureus Isolates of Lineage ST1640 and Those Carrying the lukPQ Gene. Animals.

[8] Wanapat M., Cherdthong A., Phesatcha K., Kang S. (2015). Dietary sources and their effects on animal production and environmental sustainability. Anim. Nutr..

[9] Broucek J. (2014). Production of Methane Emissions from Ruminant Husbandry: A Review. J. Environ. Prot..

[10] Johnson K.A., Johnson D.E. (1995). Methane emissions from cattle. J. Anim. Sci..

[11] Swain P.S., Dominic G., Bhakthavatsalam K.V.S., Terhuja M., Swain P.S., Dominic Á.G. (2016). Impact of Ruminants on Global Warming: Indian and Global Context. Environmental Science and Engineering.

[12] Wang W.J., Wang S.P., Luo D.M., Zhao X.L., Yin M.J., Zhou C.F., Liu G.W. (2019). Effect of Chinese herbal medicines on rumen fermentation, methanogenesis and microbial flora in vitro. S. Afr. J. Anim. Sci..

[13] Patra A.K. (2012). Dietary Phytochemicals and Microbes.

[14] AOAC (2000). Official Methods of Analysis of AOAC International.

[15] Van Soest P.J., Robertson J.B., Lewis B.A. (1991). Methods for Dietary Fiber Neutral Detergent Fiber, and Nonstarch Polysaccharides in Relation to Animal Nutrition. J. Dairy Sci..

[16] Ottenstein D.M., Bartley D.A. (1971). Improved Gas Chromatography Separation of Free Acids C2-C5 in Dilute Solution. Anal. Chem..

[17] Tilley J.M.A., Terry R.A. (1963). A two-stage technique for the in vitro digestion of forage crops. Grass Forage Sci..

[18] Engels E.A.N., Van der Merwe F.J. (1967). Application of an in vitro technique to South African forages with special reference to the effect to certain factors on the results. S. Afr. J. Agric. Sci..

[19] Ikhimioya I. (2019). Acceptability of selected common shrubs/tree leaves in Nigeria by West African Dwarf goats. Livest. Res. Rural. Dev..

[20] Taranto F., Pasqualone A., Mangini G., Tripodi P., Miazzi M.M., Pavan S., Montemurro C. (2017). Polyphenol Oxidases in Crops: Biochemical Physiological and Genetic Aspects. Int. J. Mol. Sci..

[21] Mediani A., Abas F., Tan C., Khatib A. (2014). Effects of Different Drying Methods and Storage Time on Free Radical Scavenging Activity and Total Phenolic Content of Cosmos Caudatus. Antioxidants.

[22] Alizadeh Amoli Z., Mehdizadeh T., Tajik H., Azizkhani M. (2019). Shelf life extension of refrigerated, vacuum-packed rainbow trout dipped in an alginate coating containing an ethanolic extract and/or the essential oil of Mentha Aquatica. Chem. Pap..

[23] Stafford G.I., Jäger A.K., van Staden J. (2005). Effect of storage on the chemical composition and biological activity of several popular South African medicinal plants. J. Ethnopharmacol..

[24] Amoo S.O., Aremu A.O., Moyo M., Van Staden J. (2012). Antioxidant and acetylcholinesterase-inhibitory properties of long-term stored medicinal plants. BMC Complement. Altern. Med..

[25] Forabosco F., Chitchyan Z., Mantovani R. (2017). Methane nitrous oxide emissions and mitigation strategies for livestock in developing countries: A review. S. Afr. J. Anim. Sci..

[26] Haque M.N. (2018). Dietary manipulation: A sustainable way to mitigate methane emissions from ruminants. JAST.

[27] Martinez C.M., Chung Y.-H., Ishler V.A., Bailey K.W., Varga G.A. (2009). Effects of dietary forage level and monensin on lactation performance, digestibility and fecal excretion of nutrients, and efficiency of feed nitrogen utilization of Holstein dairy cows. J. Dairy Sci..

[28] Balsamo R.A., Willigen C.V., Bauer A.M., Farrant J. (2006). Drought tolerance of selected Eragrostis species correlates with leaf tensile properties. Ann. Bot..

[29] Hundal J.S., Singh I., Wadhwa M., Singh C., Uppal C., Kaur G. (2019). Effect of Punica granatum and Tecomella undulata supplementation on nutrient utilization enteric methane emission and growth performance of Murrah male buffaloes. J. Anim. Feed Sci..

[30] Kumar Singh R., Dey A., Punia B., Paul S., Singh M., Kumar R. (2017). Supplementing blends of plant secondary metabolites as phytobiotics for modulation of in vitro methanogenesis and rumen fermentation in buffalo. Bull. Environ. Pharmacol. Life Sci..

[31] Elghandour M.M.Y., Salem A.Z.M., Khusro A., Cipriano-Salazar M., Olivares-Pérez J., Barros-Rodriguez M.A., Lugo Coyote R. (2017). Assessment of some browse tree leaves on gas production and sustainable mitigation of CH4 and CO2 emissions in dairy calves at different age. J. Clean. Prod..

[32] Faniyi T.O., Prates E.R., Adegbeye M.J., Adewumi M.K., Elghandour M.M.Y., Salem A.Z.M., Ritt L.A., Zubieta A.S., Stella L., Ticiani E. (2019). Prediction of biogas and pressure from rumen fermentation using plant extracts to enhance biodigestibility and mitigate biogases. ESPR.

[33] Linda J., Okon E.O. (2014). Comparative Study of the Phytochemical Properties of Jatropha curcas and Azadirachta indica Plant Extracts. J. Poisonous Med. Plants Res..

[34] Cieslak A., Zmora P., Pers-Kamczyc E., Szumacher-Strabel M. (2012). Effects of tannins source (Vaccinium vitis idaea L.) on rumen microbial fermentation in vivo. Anim. Feed Sci. Technol..

[35] Patra A.K., Yu Z. (2014). Combinations of nitrate saponin, and sulfate additively reduce methane production by rumen cultures in vitro while not adversely affecting feed digestion fermentation or microbial communities. Bioresour. Technol..

[36] Delgado D.C., Galindo J., Gonzalez R., Savon L., Scull I., Gonzalez N., Marrero Y. Potential of tropical plants to exert defaunating effects on the rumen and to reduce methane production. Proceedings of the Sustainable Improvement of Animal Production and Health.

[37] Harborne J.B., Williams C.A. (2000). Advances in flavonoid research since 1992. Phytochemistry.

[38] Kim E.T., Guan L.L., Lee S.J., Lee S.M., Lee S.S., Lee I.D., Lee S.K., Lee S.S. (2015). Effects of Flavonoid-rich Plant Extracts on In vitro Ruminal Methanogenesis, Microbial Populations and Fermentation Characteristics. Asian-Australas. J. Anim. Sci..

[39] Adejoro F.A., Hassen A., Akanmu A.M., Morgavi D.P. (2020). Replacing urea with nitrate as a non-protein nitrogen source increases lambs’ growth and reduces methane production, whereas acacia tannin has no effect. Anim. Feed Sci. Technol..

[40] Castro-Montoya J.M., Makkar H.P.S., Becker K. (2011). Chemical composition of rumen microbial fraction and fermentation parameters as affected by tannins and saponins using an in vitro rumen fermentation system. Can. J. Anim. Sci..

